# Daily Intake of Protein from Cod Residual Material Lowers Serum Concentrations of Nonesterified Fatty Acids in Overweight Healthy Adults: A Randomized Double-Blind Pilot Study

**DOI:** 10.3390/md16060197

**Published:** 2018-06-05

**Authors:** Iselin Vildmyren, Huy John Vu Cao, Lina Bowitz Haug, Ida Ulrikke Valand, Øyvin Eng, Åge Oterhals, Maren Hoff Austgulen, Alfred Halstensen, Gunnar Mellgren, Oddrun A. Gudbrandsen

**Affiliations:** 1 Dietary Protein Research Group, Department of Clinical Medicine, University of Bergen, 5021 Bergen, Norway; iselin.vildmyren@uib.no (I.V.); john_cao@outlook.com (H.J.V.C.); linabhaug@gmail.com (L.B.H.); ida.ulrikke.valand@uia.no (I.U.V.); maren_austgulen@hotmail.com (M.H.A.); 2 K. Halstensen AS, Box 103, 5399 Bekkjarvik, Norway; alfred.halstensen@uib.no; 3 Hormone Laboratory, Haukeland University Hospital, 5021 Bergen, Norway; Oyvin_eng@hotmail.com (Ø.E.); gunnar.mellgren@uib.no (G.M.); 4 Nofima AS, P.B. 1425 Oasen, 5828 Bergen, Norway; Aage.Oterhals@Nofima.no; 5 Department of Clinical Science, University of Bergen, 5021 Bergen, Norway

**Keywords:** glucose regulation, overweight, obesity, cod, protein supplement, residual material

## Abstract

Improved process technologies have allowed fishing vessels to utilize residuals from cod fillet production (head, backbone, skin, cuttings, and entrails) and convert this to high-quality protein powders for human consumption. In this double-blind pilot study, 42 healthy overweight or obese adults were randomized to three experimental groups consuming tablets corresponding to 6 g/day of proteins from cod residuals as presscake meal (Cod-PC), presscake and stickwater meal (Cod-PCW), or placebo tablets (control) for eight weeks. The primary outcome of this study was changes in metabolites related to glucose regulation in overweight or obese healthy adults after intake of proteins from cod residuals. Cod-PC supplementation decreased postprandial serum nonesterified fatty acids (NEFA) concentration and increased gene expressions of diglyceride acyltransferase 1 and 2 in subcutaneous adipose tissue compared with controls. Fasting insulin increased while fasting NEFA and 120-min postprandial glucose decreased within the Cod-PC group, but these changes did not differ from the other groups. In conclusion, supplementation with Cod-PC beneficially affected postprandial serum NEFA concentration compared with the other groups in overweight or obese adults. Supplementation with Cod-PCW, which contains a higher fraction of water-soluble protein compared to Cod-PC, did not affect serum markers of glucose regulation.

## 1. Introduction

Being overweight or obese is becoming increasingly prevalent worldwide and no country to date has been able to reverse this development [1]. Such conditions, especially obesity, are associated with increased risk of developing impaired glucose tolerance, which can lead to type 2 diabetes mellitus and premature mortality [2]. Impaired glucose tolerance involves elevated levels of glucose, insulin, and nonesterified fatty acids (NEFAs) in the circulation [3]. Therefore, lowering the concentrations of these risk markers should be targeted as a measure to prevent disease development in overweight and obese adults.

Fish consumption as part of a balanced diet has been associated with beneficial health effects [4,5], and recent studies have shown that cod intake can improve glucose regulation and insulin sensitivity in both rats and humans [6,7,8,9]. Since cod is a lean fish, these findings suggest that not only the long-chain *n*-3 polyunsaturated fatty acids (PUFAs) may be important in explaining positive health effects derived from fish intake, but also other nutrients such as proteins may be beneficial.

Intervention studies using cod have thus far focused mainly on health effects of the fillet, which accounts for approximately 50–70% of the whole-fish dry weight [10]. The remaining 30–50% of the whole fish is referred to as residual material and consists of head, backbone, skin, cuttings, and entrails, which should be considered as a valuable source of marine proteins, with levels up to 70% of the dry matter. Improved processing technologies allow more of the residual material to be processed immediately on-board the fishing vessels, thus retaining its high quality and conserving a greater extent of the proteins which otherwise would be discarded [11]. These high-quality protein powders from residual materials can be utilized for human consumption and thereby provide economic benefits for the industry.

The present study is, to the best of our knowledge, the first to investigate effects of protein-rich residual materials from cod in humans. The aim of this study was to investigate the effect of protein intake from cod residual material on glucose regulation in overweight or obese adults. We hypothesized that eight weeks of supplementation with 6 g of cod residual protein per day, i.e., presscake meal with a low content of water-soluble protein (Cod-PC) or presscake and stickwater meal with a high content of water-soluble protein (Cod-PCW), would improve markers of glucose regulation in adults who are overweight or obese.

## 2. Results

### 2.1. Participant Characteristics

**Figure 1:**
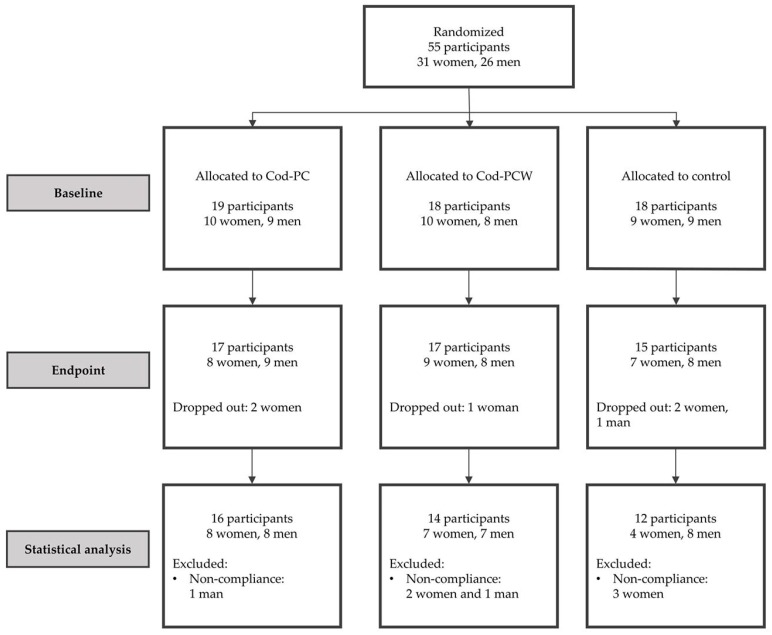
Cod-PC, cod presscake meal; Cod-PCW, cod presscake and stickwater meal. Flow diagram displaying the progress of participants during the study period. Participants who did not comply with the study protocol were excluded from statistical analysis. Noncompliance was defined as not following the protocol in regard to fish intake, other dietary changes (including dietary supplements), and newly discovered diseases or use of prescription medicine not compatible with the inclusion criteria.

Fifty-five participants were randomized to three intervention groups, and of these, 42 participants completed the intervention according to the study protocol. Six participants (11%) withdrew from the study (two from the Cod-PC group, one from the Cod-PCW group, and three from the control group). Seven participants (13%) were excluded from statistical analysis due to noncompliance (one in the Cod-PC group, three in the Cod-PCW group, and three in the control group). Noncompliance was defined as not following study protocol including failing to take the intervention supplements, initiating use of medications, disease outbreak, or implementing dietary changes which could implicate the study outcome. The flow of participants in the study is presented in Figure 1. 

Age, body mass index (BMI), body weight, body fat percentage, and use of tobacco were similar between groups at baseline (Table 1). BMI, body weight, and body fat percentage did not change from baseline to endpoint in any of the intervention groups (data not presented). All participants had HbA1c (glycated haemoglobin) levels within normal <6.0% (42 mmol/mol IFCC) at baseline.

### 2.2. Estimated Energy and Macronutrients Intake and Physical Activity

Estimated energy and macronutrients intake and reported physical activity were similar between the groups at baseline. Energy intake was increased within the Cod-PC group from baseline to endpoint, whereas no change was seen in the Cod-PCW group or in the control group, and changes were similar between groups when compared (Table 2). Fat intake (as percentage of energy intake) decreased within the Cod-PCW group, but no change was seen within the other intervention groups. The decrease in fat intake in the Cod-PCW group was significantly different from changes within the Cod-PC group but not when compared with the control group. Physical activity was not changed within or between groups from baseline to endpoint.

### 2.3. Concentrations of Circulating Markers of Glucose Regulation

Circulating concentrations of fasting and postprandial glucose, NEFA, insulin, and glucagon-like peptide-1 (GLP-1) were similar between the three intervention groups at baseline. Concentrations of fasting glucose and GLP-1 were not changed within groups or between groups from baseline to endpoint. Fasting serum NEFA decreased and fasting serum insulin increased within the Cod-PC group from baseline to endpoint, whereas no change was seen within the other intervention groups and changes were similar between all groups (Table 3). Serum glucose concentration 120 min after the standardized breakfast meal decreased within the Cod-PC group after eight weeks, but this change was not different from the other groups. 

**Figure 2:**
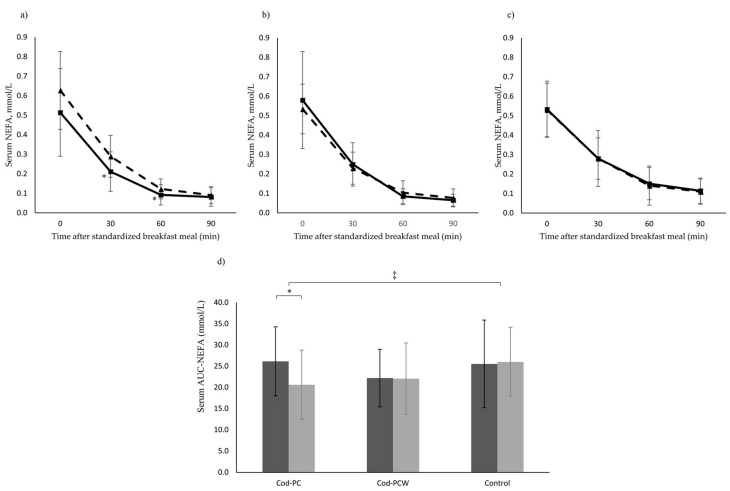
Cod-PC, cod presscake meal; Cod-PCW, cod presscake and stickwater meal. NEFA response after intake of a standardized breakfast meal in the Cod-PC group (**a**), Cod-PCW group (**b**), the control group (**c**), and AUC-NEFA for all groups (**d**). NEFA concentrations were measured at baseline (dotted line) and after eight weeks (solid line). Data are presented as mean and standard deviation. Postprandial NEFA concentrations are presented for 16 participants in the Cod-PC group, 12 participants in the Cod-PCW group, and 12 participants in the control group. * Changes within groups were measured using a paired sample *T*-test. ^‡^ Changes within the Cod-PC group were compared with the Cod-PCW group and the control group using one-way ANOVA followed by LSD as post hoc. *p*-value < 0.05 was considered significant.

Areas under the curve (AUC)-NEFA and NEFA concentrations 30 and 60 min post breakfast meal decreased within the Cod-PC group, but there were no changes within the other intervention groups from baseline to endpoint (Figure 2). AUC-NEFA in the Cod-PC group decreased compared with changes in the Cod-PCW group and in the control group, postprandial 30 min NEFA concentration decreased in the Cod-PC group compared with changes in the Cod-PCW group, and postprandial 60 min NEFA concentration decreased in the Cod-PC group compared with changes in the control group (Figure 2). AUC-glucose, postprandial glucose concentrations (30, 60, and 90 min post breakfast meal), AUC-insulin, postprandial insulin concentrations (30, 60, 90, and 120 min post breakfast meal), and postprandial GLP-1 concentrations (30 and 60 min post breakfast meal) did not change between groups from baseline to endpoint (data not shown). 

### 2.4. mRNA Expressions in Subcutaneous Adipose Tissue

The mRNA expressions were measured in subcutaneous adipose tissue extracted via needle biopsy. Genes were selected to explore NEFA regulation in adipose tissue in relation to NEFA levels in the circulation and because diglyceride acyltransferase (DGAT) 1 and DGAT2 are associated with insulin sensitivity. The mRNA expressions of DGAT1, DGAT2, and Lipoprotein lipase (LPL) increased within the Cod-PC group from baseline to endpoint, with no changes within the other intervention groups (Table 4). The expression of DGAT1 and DGAT2 was significantly increased in the Cod-PC group compared with the control group, but the change in LPL expression over time was similar between all groups. The gene expression of cluster-determinant 36 molecule (CD36) was not changed within any of the groups or between groups from baseline to endpoint.

## 3. Discussion

The main findings of the current study are that supplementation with protein from Cod-PC decreased postprandial serum NEFA concentrations and increased gene expressions of DGAT1 and DGAT2 in subcutaneous adipose tissue in overweight or obese adults. The current study shows that protein from residual material from cod may affect indicators of human health and could encourage the industry to regard coproducts from fish-fillet production as nutritionally beneficial. To our knowledge, this is the first study to investigate the health effects of cod protein residual material intake.

Studies exploring effects on glucose regulation with cod consumption with either cod fillet intake or cod protein supplementation have come to variable conclusions depending on study design and population [6,7,8,9,12,13,14]. Intake of cod fillet has been shown to improve glucose regulation in insulin resistant individuals [7], however a high cod intake (750 g/week) seems to have little impact on glucose and insulin concentrations in normoglycemic normal-weight and overweight adults [13,14]. Isolated proteins from cod muscle, on the other hand, beneficially improved postprandial glucose concentrations in both nondiabetic humans and rats [6,8,9,12]. The decreased postprandial NEFA seen in the Cod-PC group in the current study could imply effects on glucose regulation seen together with the decrease in 2-h postprandial glucose. Both high postprandial glucose and NEFA concentrations are associated with reduced glucose tolerance [3,15], and lower concentrations of both these markers have been observed when rats were fed cod muscle hydrolysate [9]. The observed increase in fasting insulin concentration together with no change in fasting glucose within the Cod-PC group could suggest reduced insulin sensitivity. However, there was also a decrease in fasting NEFA concentration within the Cod-PC group, which is a strong regulator of insulin sensitivity, and NEFA concentration is inversely correlated with insulin resistance [3].

The higher gene expression of DGAT1 and DGAT2 observed in adipose tissue after Cod-PC supplements in the current study also implies an improvement in glucose regulation, as these genes are positively associated with insulin sensitivity, and can be elevated in adults with normal glucose tolerance compared with adults with impaired glucose tolerance [16]. It is unclear how proteins from cod residuals would increase expression of the DGAT genes, but there is some evidence to suggest that DGAT is stimulated by insulin [17], which was increased in the Cod-PC group. Increased expression of DGAT1 and DGAT2 also fits in with the decreased serum NEFA concentration observed in the Cod-PC group, suggesting that more NEFA was cleared from the circulation and included in production of triacylglycerols in subcutaneous adipose tissue. 

The branched chain amino acids (BCAA) leucine, isoleucine and valine [8], arginine [18], and taurine [19] found in both cod protein supplements in the current study have shown beneficial effects or have been discussed in relation to improved glucose regulation [8,18,19]. Thus, it is possible that the higher intake of BCAA from the Cod-PC tablets compared with the Cod-PCW tablets may partly explain the reduced postprandial glucose and NEFA concentrations in the Cod-PC group, as BCAA intake has been shown to improve glucose regulation in healthy individuals [20]. Given that the arginine intake from the tablets was comparable between the two cod protein supplements and the taurine intake was higher from the Cod-PCW tablets, the effects on glucose and NEFA in the Cod-PC group are not likely attributed to arginine and taurine intake from the intervention supplements. The daily contribution of the essential fatty acids 18:2*n*-6 and 18:3*n*-3 and the long-chain *n*-3 PUFAs 20:5*n*-3, 22:5*n*-3, and 22:6*n*-3 were found in slightly higher amounts in the Cod-PC tablets compared to the Cod-PCW tablets, but given that the intake is so low compared with doses necessary to see effects in n-3 PUFA supplement studies [21,22,23], we believe it is not likely that such low intakes of n-3 PUFAs would affect markers of glucose regulation in the current study. An alternative explanation is that bioactive motifs may be responsible for the observed reduction in postprandial NEFA in the Cod-PC group compared to control and the reduced postprandial glucose within the Cod-PC group. Bioactive peptides are described as peptides with specific physiological effects, either locally in the digestive tract or in other organs which they target through the circulatory system [24]. Bioactive motifs with dipeptidyl peptidase-4 inhibitor properties, such as isoleucine-prolin-isoleucin (IPI), have been found in residual material from other marine fish species (herring and salmon) [25] and may also be present in residual material from cod in the current study. IPI is described as the most potent dipeptidyl peptidase-4 inhibitor [26] and indirectly promotes insulin secretion, which in turn inhibits lipolysis in adipose tissue. The increased serum insulin within the Cod-PC group in the current study could, if IPI was present in the cod residuals, partially be explained by the dipeptidyl peptidase-4 inhibitor actions of this bioactive motif. In addition, with the inhibitory effect of insulin on lipolysis, the increased insulin concentration in the Cod-PC group could explain the reduction in circulating NEFA.

There are some limitations to the current study. The sample size calculations were difficult to perform as we had no similar studies with cod residual intake with which to compare. Therefore, we cannot exclude the possibility of effects not being discovered (type 2 error) as a consequence of small sample size. As a pilot, the current study can be a valuable contribution in the planning and sample size calculations for future studies with cod residual material. We observed that the estimated total energy intake was increased within the Cod-PC group, however, this was not accompanied by changes in intake of the individual macronutrients and also no increase was seen in body weight from baseline to endpoint.

The findings in the current study are partially in line with our hypothesis that eight weeks of supplementation with 6 g/day of cod protein would beneficially affect circulating markers of glucose regulation in overweight or obese adults, as we found that postprandial NEFA concentration was decreased after eight weeks of Cod-PC supplements. Future studies should consider including adults with impaired glucose tolerance and explore whether a higher daily dose of protein from cod residual material would give more profound effects on glucose regulation in these individuals.

## 4. Materials and Methods 

### 4.1. Participants, Study Design, and Ethics

The present study included healthy overweight or obese adults living in Bergen and the surrounding areas. Participants were recruited through advertising in the internal network at Haukeland University Hospital (Bergen, Norway) and posters (Bergen city centre and at Haukeland University Hospital). Recruitment took place in August and September 2013, and the study was conducted in September, October, and November 2013. 

Eligibility criteria for participation in the study were age between 20 and 65 years, BMI >28 kg/m^2^, stable body weight with less than 5 kg fluctuation during the preceding 4 months, and fasting blood glucose <7.0 mmol/L. Exclusion criteria were known diseases or metabolic disturbances related to being overweight or obese, use of prescription medications that affect blood lipids or blood glucose concentrations, allergies towards fish or seafood, undertaking a weight loss diet, or tobacco use exceeding >15 cigarettes or snus/day. Pregnant or lactating women and candidates with a seafood consumption exceeding 200 g/week or taking dietary supplements including cod liver oil or other marine fatty acids were not included in the study.

Fifty-five eligible volunteers were included in this randomized, parallel, double-blind intervention study conducted at the University of Bergen (Bergen, Norway). Participants were stratified according to gender, age, and BMI before randomization to one of the three experimental groups. The intervention tablets were coded (A, B, and C) by the manufacturer to ensure that all participants and personnel in the study were blinded. The study was conducted in accordance with the guidelines laid down by the Declaration of Helsinki and was approved by the Regional Ethics Committee of Western Norway (approval No.: 2011/572). All participants provided written informed consent before enrolment in the study. The study is registered at clinical trials.gov (NCT01894542).

### 4.2. Intervention and Protocol for Study Visits

Participants consumed 6 g of cod protein or control supplements daily for eight weeks (18 tablets per day). During the study period, participants were instructed to maintain their normal dietary habits and level of physical activity. Both intervention supplements contained fishmeal of residual material from Northeast Atlantic cod *(Gadus morhua).* Participants in the three intervention arms received either supplements containing protein from Cod-PC, supplements containing protein from Cod-PCW, or control supplements with no active ingredients (placebo). 

Participants attended two study visits during the intervention period; one visit at baseline and one visit at endpoint, i.e., after eight weeks. Both study visits were conducted after an overnight fast (>10 h) which included no intake of food or drink except water and no use of tobacco or medications. Participants were advised to avoid intensive physical activity and alcohol intake during the 24 h preceding each study visit. Prior to the baseline visit, all participants received an e-mail with information about the study, the declaration of consent, and the forms to fill in their dietary records.

During the baseline visit, fasting blood glucose was measured using the Contour blood glucose meter (Bayer Consumer Care AG, Basel, Switzerland) to ensure that the participants’ fasting blood glucose was <7 mmol/L. Height was measured using a stadiometer (MZ10023-3, ADE GmbH & Co., Hamburg, Germany). Body composition was measured at the baseline and the endpoint visits using a bioelectrical impedance analyser (Tanita BC-418 Segmental Body Composition Analyzer, Arlington Heights, IL, USA) while participants were in a fasting state. Blood samples were collected at baseline and endpoint using an antecubital venous catheter. Fasting blood samples were drawn in the morning between 8:00 and 10:00 a.m., and postprandial samples were drawn after consuming a standardized breakfast meal. Blood samples were collected in Vacuette Z Serum Clot Activator Tubes (Greiner Bio-One, Frickenhausen, Germany) for isolation of serum and Vacuette K2EDTA (Greiner Bio-One GmbH, Germany) for isolation of plasma. GLP-1 was preserved in EDTA blood samples by immediately adding 10 μL dipeptidyl peptidase-IV Inhibitor (DRG Instruments GmbH, Marburg, Germany) per mL whole blood. Serum, plasma, and whole blood samples were stored at −80 °C until analysis.

The standardized breakfast meal consisted of two crisp breads (Wasa Frukost, Barilla AS, Stockholm, Sweden), 20 g margarine (Soft Flora, Mills DA, Oslo, Norway), 40 g cheese (Gräddost, Tine AS, Oslo, Norway), 4 crackers (Gjende original, Orkla Confectionery & Snacks, Solna, Sweden), and a drink containing 0.06 L fruit concentrate (Husholdningssaft, Rema 1000, Oslo, Norway) and 20 g glucose powder (Nutana, Mariager, Denmark) mixed with 0.25 L water. The total energy content of the standardized breakfast meal was 739 kcal and contained 13 g protein, 37 g fat, 87 g carbohydrate, and 3 g fibre. The participants were instructed to consume the breakfast meal within 10 min at the first visit and were encouraged to eat at similar pace during the second visit.

The subcutaneous adipose tissue biopsy was performed in the lower abdominal region of all participants, however, the quality of three samples (one from each group) was too poor for further analysis and the samples were discarded. The biopsy was initiated by applying an anaesthetic EMLA patch (Aspen Pharmacare, St Leonards NSW, Australia) on the biopsy area approximately 45 min before the biopsy, followed by a local anaesthetic injected in a fan shape (5 μL Lidokain 10 mg/mL FarmaPlus AS, Oslo, Norway). An incision (2–3 mm) was made using a scalpel, then a Hepafix syringe (Hepafix^®^ from B.Braun, Melsungen AG, Melsungen, Germany) with a Sterican biopsy needle (B.Braun, Melsungen AG) was inserted and subcutaneous adipose tissue was extracted. The adipose tissue sample was immediately rinsed twice with sterile 0.9% NaCl (Ecoflac^®^, B.Braun, Melsungen AG) solution before it was transferred to liquid nitrogen. Biopsy samples were stored at −80 °C until analysis.

### 4.3. Production, Analyses, and Contents of Intervention Tablets

Cod residual material from cod fillet production was processed on-board the fishing vessel Granit (Halstensen Granit AS, Bekkjarvik, Norway). The fish residuals were grinded and heat treated in a continuous cooker before mechanical pressing to separate most of the aqueous fraction (stickwater) containing water-soluble protein from the solid phase (presscake). The solid phase was dried onboard the fishing vessel to produce a presscake meal, while the stickwater was frozen and transported to land where it was thawed and concentrated before mixed with the presscake meal and dried at Nofima (Bergen, Norway). Two fishmeals were produced for inclusion in the supplements. The Cod-PC tablets contained a meal based on cod presscake as the sole source of protein, containing only remnants of water-soluble protein corresponding to 15.5% of crude protein, and the Cod-PCW tablets contained a meal based on presscake with the addition of water-soluble protein in the form of stickwater, corresponding to 35.4% of crude protein.

All intervention tablets contained similar amounts of sorbitol, tricalcium phosphate, and magnesium stearate. The cod protein supplements contained fishmeal from cod residual material (Table 5). Microcrystalline cellulose was used as a filler and replaced fishmeal in the control supplements. Tablets were produced by Faun Pharma AS (Vestby, Norway). All analyses of the intervention tablets were conducted by Nofima BioLab (Bergen, Norway). Ten tablets from each supplement were crushed and mixed, and two samples from each supplement were taken for each analysis. The presented results are the means of two measurements. The microorganism Salmonella was analysed using PCR combined with NMKL method 71 [27] and aerobe microorganisms were analysed with Petrifilm™ Aerobic Count Plate (ISO 4833-1). Enterobacteriaceae were analysed using the colony count technique (ISO 21528-2), Coliform bacteria and Escherichia coli were analysed using Petrifilm™ Coliform/Escherichia coli Count Plate (NordVal 014), and Presumptive Bacillus cereus was analysed using the colony count technique (ISO 7932). Mould and yeast were analysed with Petrifilm™ Mold and Yeast Count plate (NordVal 016). The crude protein content (N × 6.25) of the supplements was determined by the Dumas method (AOAC 990.03). Water-soluble protein content was determined based on the Kjeldahl method (ISO 5983-2). Total amino acid composition was measured by HPLC according to the method of Cohen and Michaud [28]. Tryptophan was determined by the method of Miller [29]. Taurine was measured in the water-soluble protein by HPLC using the Waters Pico-Tag method [30]. Fat content and fatty acid composition was prepared according to the AOCS method Ce 1b-89 and measured as previously described by Oterhals and Nygård [31].

The daily intake of the amino acids arginine, histidine, isoleucine, lysine, methionine, phenylalanine, threonine, tryptophan and valine from intervention tablets were comparable in the Cod-PC and Cod-PCW tablets (i.e., less than 40 mg/day difference), whereas the intake of leucine was higher from the Cod-PC tablets compared with the Cod-PCW tablets, and glycine and taurine from the Cod-PCW tablets were higher than from the Cod-PC tablets (Table 6). The essential fatty acids 18:2n-6 and 18:3n-3 and the long-chain n-3 PUFAs were found in slightly higher amounts in the Cod-PC tablets compared to the Cod-PCW tablets, whereas the amounts of these fatty acids were below detection levels in the control tablets (Table 6).

### 4.4. Estimation of Energy and Macronutrient Intakes from Dietary Records, and Estimation of Physical Activity

Participants recorded their dietary intake the five days preceding the baseline and endpoint visits. The dietary records were used to estimate the participants’ intake of energy and macronutrients using the dietary assessment software Mat på Data 5.1 (Norwegian Food Safety Authority, Oslo, Norway), which is based on the Norwegian Food Composition Table (Norwegian Food Safety Authority, Oslo, Norway). The protein content from the intervention tablets was added to the participants’ dietary records at the endpoint visit. Physical activity was calculated based on the Borg rating of perceived exertion scale [32] and participants registered their physical activities for the two weeks before the baseline visit and before the endpoint visit.

### 4.5. Serum Analyses

Analyses of serum glucose were performed by accredited routine methods at the Laboratory of Clinical Biochemistry at Haukeland University Hospital (Bergen, Norway). Serum NEFA was analysed on the Cobas c 111 system (Roche Diagnostics GmbH, Marburg, Germany) using NEFA FS kit (Diagnostic Systems, Holzheim, Germany). HbA1c in whole blood was analysed on the Cobas c 111 system using the A1C-3 kit (Roche Diagnostics GmbH, Marburg, Germany). Serum insulin was analysed using the ELISA kit EIA-2935 (DRG Instruments GmbH, Marburg, Germany). Plasma GLP-1 (active) was analysed using the ELISA kit EIA-3065 (DRG Instruments GmbH, Marburg, Germany). AUC for glucose, NEFA, and insulin were calculated using the trapezoid formula [33].

### 4.6. Analyses of mRNA Expression in Subcutaneous Adipose Tissue 

Total RNA was purified from subcutaneous white adipose tissue using the RNeasy Mini Kit (Qiagen, Hilden, Germany) according to the manufacturer’s protocol. RNA concentration and quality were measured using QIAxpert (Qiagen, Hilden, Germany). High Capacity cDNA Reverse Transcription Kit (Applied Biosystems, CA, USA) was used to synthesize cDNA from 150 ng total RNA per sample. cDNA was diluted 1:5 with PCR-grade water before qPCR was performed (in triplicate) using the LightCycler480 rapid thermal cycler system (Roche Diagnostics GmbH, Basel, Switzerland) with the LightCycler 480 SYBR Green I Master (Roche, Basel, Switzerland). The following primers were used: DGAT1; forward primer 3′actaccgtggcatcctgaac’5 and reverse primer 3′ataaccgggcattgctca’5, DGAT2; forward primer 3′actaccgtggcatcctgaac’5 and reverse primer 3′ataaccgggcattgctca’5, LPL; forward primer 3′caggcctttgagatttctctgt’5 and reverse primer 3′gaaggagtaggtcttatttgtggaa’5, CD36; forward primer 3′tggaacagaggctgacaactt’5 and reverse primer 3′ttgattttgatagatatgggatgc’5. As reference genes, 3 primer pairs were tested: Hypoxanthine phosphoribosyltransferase 1 (HPRT1); forward primer 3′tgaccttgatttattttgcatacc’5 and reverse primer 3′cgagcaagacgttcagtcct’5, importin 8 (IPO8); forward primer 3′acagcactgcaggaggtgta’5 and reverse primer 3′gcctccctgttgttcaatct’5, TATA-box binding protein (TBP) forward primer 3′tgaatcttggttgtaaacttgacc’5 and reverse primer 3′ctcatgattaccgcagcaaa’5. Of these reference genes, IPO8 had least variation in readings of samples in triplets, therefore results are presented relative to IPO8.

### 4.7. Outcome Measurements

The primary outcome of this study was changes in metabolites related to glucose regulation in overweight or obese adults after eight weeks supplementation with proteins from cod residual material with either a low or high content of water-soluble protein. The secondary outcomes were changes in gene expression in adipose tissue related to circulating markers of glucose regulation.

### 4.8. Sample Size

Health effects of supplementation with cod residual material has not previously been studied in overweight or obese adults and the current study is therefore considered a pilot. A previous study by Vikøren et al. [8] observed that 3 g (first four weeks) followed by 6 g (last four weeks) per day of cod fillet proteins affected serum glucose concentrations after a standardized breakfast meal in 18 overweight or obese adults. In the present study, the daily protein intake from supplements was 6 g, and the study population was overweight or obese adults (BMI > 28 kg/m^2^), i.e., similar to that of Vikøren et al. However, while the current study used cod residual material, the previous study used cod fillet and, hence, we cannot use the previous study to calculate sample size. We therefore consider the current study to be hypothesis generating instead of hypothesis testing, and findings in the present study will form the basis for sample-size calculations for future studies with a similar design.

### 4.9. Statistical Analyses

Statistical analyses were performed by using PASW Statistics version 25 (IBM Corp. Released 2017. IBM SPSS Statistics for Windows, Version 25.0. IBM Corp., Armonk, NY, USA). All variables were evaluated for normality by the Shapiro–Wilk test, Q-Q plots and histograms. Most variables were normally distributed, and the remaining variables were log-transformed before performing the parametric statistical tests. Changes within groups from baseline to endpoint were tested using the paired sampled *T*-test. The one-way-ANOVA was used to compare changes between the Cod-PC, Cod-PCW, and control group, and the least significant difference (LSD) test was applied as the post hoc test when appropriate. Level of significance was set to *p* < 0.05.

## 5. Conclusions

In conclusion, supplementation with Cod-PC had a beneficial effect on postprandial NEFA concentration in overweight or obese adults. The observation that fasting insulin was higher after Cod-PC supplements, whereas fasting glucose was unchanged, suggests reduced insulin sensitivity. However, since fasting and postprandial NEFA and postprandial 120-min glucose were decreased, this indicates that insulin sensitivity was not reduced after Cod-PC intake. Supplementation with Cod-PCW, however, which contains a higher fraction of water-soluble protein when compared to Cod-PC, did not lead to changes in the investigated serum markers of glucose regulation. These findings suggest that consuming presscake meal alone is more advantageous than consuming presscake meal added high amounts of stickwater. As a hypothesis generating pilot study, the current results should encourage new studies to further elucidate the possible health effects of protein from cod residual material.

## Figures and Tables

**Table 1:** Participant characteristics at baseline.

	Cod-PC (*n* = 16)		Cod-PCW (*n* = 14)		Control (*n* = 12)		*p*
Characteristics	Mean	SD	Mean	SD	Mean	SD	
Women/Men	8/8		7/7		4/8		0.62
Age (years)	40.1	12.7	45.0	12.0	41.1	11.1	0.52
BMI (kg/m^2^)	33.3	4.4	34.2	5.0	35.0	4.7	0.86
Body weight (kg)	100.6	15.6	104.5	16.2	108.0	17.1	0.49
Fat (%)	35.6	7.9	37.2	10.4	35.1	9.1	0.77
Smoke/snus *	3		2		3		0.89

Cod-PC, cod presscake meal; Cod-PCW, cod presscake and stickwater meal; BMI, body mass index. Results are presented as mean and standard deviation; * Snus is a Scandinavian tobacco substance which is placed under the upper lip. Groups were compared at baseline using Pearson’s chi-squared test for categorical data and one-way ANOVA for continuous data. *p*-value < 0.05 was considered significant.

**Table 2:** Estimated daily dietary intake of energy and macronutrients (as percentage of energy intake).

	Baseline		8 Weeks		*p* ^†^	*p* ^‡^	*p* ^§^
	Mean	SD	Mean	SD			
Energy intake, kcal						0.11	
Cod-PC	2111	829	2422	798	0.011		
Cod-PCW	2002	335	2239	576	0.068		
Control	2033	602	2008	491	0.94		
Protein ^1^, E%						0.30	
Cod-PC	17	3	18	3	0.65		
Cod-PCW	18	2	16	3	0.12		
Control	18	3	18	3	0.77		
Fat, E%						0.044	
Cod-PC	38	11	39	7	0.21		0.22 ^A^
Cod-PCW	40	7	36	5	0.037		0.24 ^B^
Control	38	3	37	5	0.66		0.013 ^C^
Carbohydrates, E%						0.20	
Cod-PC	40	10	38	9	0.30		
Cod-PCW	39	8	40	6	0.36		
Control	37	5	39	5	0.24		
Dietary fibre, g						0.56	
Cod-PC	19	5	21	8	0.38		
Cod-PCW	19	5	19	4	0.64		
Control	17	4	18	5	0.56		

Cod-PC, cod presscake meal; Cod-PCW, cod presscake and stickwater meal. Data presented as mean and standard deviation. The groups were similar at baseline (one-way ANOVA). Estimated energy intake is presented for 16 participants in the Cod-PC group, 14 participants in the Cod-PCW group, and 12 participants in the control group. Energy intake and fat E% were not normally distributed and were log-transformed prior to testing. ^†^ Within-group changes were tested using a paired sample *T*-test. ^‡^ Within-group changes were compared between groups using one-way ANOVA. ^§^ Changes within the Cod-PC group were compared with the control group (^A^), changes within the Cod-PCW group were compared with the control group (^B^), and changes within the Cod-PC group were compared with the Cod-PCW group (^C^) using the post hoc test least significant difference (LSD). *p*-value < 0.05 was considered significant. ^1^ Protein content from the intervention tablets was added to the estimated dietary intake before the eight-week visit.

**Table 3:** Concentrations of circulating glucose, nonesterified fatty acids, insulin, and GLP-1.

	Baseline		Week 8		*p* ^†^	*p* ^‡^
Parameters	Mean	SD	Mean	SD		
Glucose (mmol/L)						0.56
Cod-PC	5.4	0.5	5.4	0.5	0.48	
Cod-PCW	5.2	0.4	5.3	0.4	0.55	
Control	5.7	0.7	5.6	0.7	0.42	
Glucose 120 min (mmol/L)						0.29
Cod-PC	5.1	1.0	4.7	1.1	0.022	
Cod-PCW	4.8	0.7	4.8	0.7	0.90	
Control	5.5	0.8	5.4	0.9	0.66	
NEFA (mmol/L)						0.065
Cod-PC	0.6	0.2	0.5	0.2	0.017	
Cod-PCW	0.5	0.1	0.6	0.2	0.48	
Control	0.5	0.1	0.5	0.1	0.93	
Insulin (pmol/L)						0.053
Cod-PC	85.8	35.6	97.0	36.8	0.014	
Cod-PCW	92.0	37.7	92.0	37.7	0.99	
Control	111.8	54.9	109.6	61.0	0.49	
Insulin 120 min (pmol/L)						0.95
Cod-PC	315.8	159.5	311.3	180.2	0.88	
Cod-PCW	270.8	144.7	278.9	136.1	0.83	
Control	417.7	264.1	409.3	229.9	0.86	
GLP-1 (pmol/L)						0.90
Cod-PC	2.9	1.0	3.1	0.7	0.15	
Cod-PCW	2.7	0.7	3.0	0.7	0.17	
Control	3.2	1.0	3.6	1.3	0.11	

Cod-PC, cod presscake meal; Cod-PCW, cod presscake and stickwater meal; NEFA, nonesterified fatty acids; GLP-1, glucagon-like peptide-1. Data presented as mean and standard deviation. The groups were similar at baseline (one-way ANOVA). Fasting serum concentrations are presented for 16 participants in the Cod-PC group, 14 participants in the Cod-PCW group, and 12 participants in the control group. Insulin and GLP-1 were not normally distributed and were log-transformed prior to testing. ^†^ Within-group changes were tested using a paired sample *T*-test. ^‡^ Within-group changes were compared between groups using one-way ANOVA. *p*-value < 0.05 was considered significant.

**Table 4:** Gene expressions in adipose tissue presented relative to IPO8 as reference gene.

	Baseline		Week 8		*p* ^†^	*p* ^‡^	*p* ^§^
mRNA	Mean	SD	Mean	SD			
DGAT1						0.02	
Cod-PC	2.14	0.52	2.55	0.63	0.0012		0.0059 ^A^
Cod-PCW	2.25	0.49	2.36	0.44	0.24		0.20 ^B^
Control	2.29	0.49	2.25	0.73	0.54		0.11 ^C^
DGAT2						0.037	
Cod-PC	2.02	0.48	2.45	0.65	0.0012		0.012 ^A^
Cod-PCW	2.15	0.61	2.27	0.51	0.16		0.25 ^B^
Control	2.20	0.53	2.2	0.82	0.71		0.14 ^C^
LPL						0.21	
Cod-PC	0.99	0.25	1.11	0.23	0.014		
Cod-PCW	1.01	0.29	1.04	0.19	0.42		
Control	1.05	0.27	1.05	0.41	0.68		
CD36						0.36	
Cod-PC	1.10	0.26	1.18	0.29	0.23		
Cod-PCW	1.10	0.26	1.18	0.29	0.43		
Control	1.25	0.33	1.22	0.34	0.50		

Cod-PC, cod presscake meal; Cod-PCW, cod presscake and stickwater meal; DGAT1, Diglyceride acyltransferase 1; DGAT2, Diglyceride acyltransferase 2; LPL, Lipoprotein lipase; CD36, Cluster-determinant 36 molecule. Data presented as mean and standard deviation. The groups were similar at baseline (one-way ANOVA). Gene expressions in adipose tissue are presented for 15 participants in the Cod-PC group, 13 participants in the Cod-PCW group, and 11 participants in the control group. None of the gene expressions were normally distributed and underwent log-transformation prior to testing. ^†^ Within-group changes were tested using a paired sample *T*-test. ^‡^ Within-group changes were compared between groups using one-way ANOVA. ^§^ Changes within the Cod-PC group were compared with the control group (^A^), changes within the Cod-PCW group were compared with the control group (^B^), and changes within the Cod-PC group were compared with the Cod-PCW group (^C^) using the post hoc test LSD. *p*-value < 0.05 was considered significant.

**Table 5:** Tablet content.

mg/Tablet	Cod-PC	Cod-PCW	Control
Residual material	522	522	0
Sorbitol	209	209	209
Microcrystalline cellulose	209	209	641
Tricalcium phosphate	10	10	10
Magnesium stearate	10	10	10
Total tablet weight	960	960	870

Cod-PC, cod presscake meal; Cod-PCW, cod presscake and stickwater meal.

**Table 6:** Amino acid, taurine, and fatty acid contents in daily dosage of cod supplements ^1^.

mg/Day	Cod-PC	Cod-PCW	Control
Amino acids			
Arginine	380.2	380.2	<LOD
Glycine	501.1	639.4	<LOD
Histidine	126.1	108.9	<LOD
Isoleucine	241.9	207.4	<LOD
Leucine	432.0	362.9	<LOD
Lysine	449.3	414.7	<LOD
Methionine	190.1	169.3	<LOD
Phenylalanine	224.6	190.1	<LOD
Taurine	120.0	230.0	<LOD
Threonine	259.2	241.9	<LOD
Tryptophan	86.4	60.5	<LOD
Fatty acids			
18:2n-6	7.9	6.2	<LOD
18:3n-3	2.4	1.4	<LOD
20:5n-3	31.0	24.9	<LOD
22:5n-3	3.2	2.8	<LOD
22:6n-3	53.3	41.5	<LOD

Cod-PC, cod presscake meal; Cod-PCW, cod presscake and stickwater meal; LOD, level of detection. ^1^ Based on the means of two measurements where deviations were less than 5% between parallels. Of the nonessential amino acids, only glycine and arginine were included in the table.
